# Crystal structure and DNA cleavage mechanism of the restriction DNA glycosylase R.CcoLI from *Campylobacter coli*

**DOI:** 10.1038/s41598-020-79537-y

**Published:** 2021-01-13

**Authors:** Ken-ichi Miyazono, Delong Wang, Tomoko Ito, Masaru Tanokura

**Affiliations:** Department of Applied Biological Chemistry, Graduate School of Agricultural and Life Sciences, The University of Tokyo, 1-1-1 Yayoi Bunkyo-ku, Tokyo, 113-8657 Japan

**Keywords:** DNA restriction-modification enzymes, X-ray crystallography

## Abstract

While most restriction enzymes catalyze the hydrolysis of phosphodiester bonds at specific nucleotide sequences in DNA, restriction enzymes of the HALFPIPE superfamily cleave *N*-glycosidic bonds, similar to DNA glycosylases. Apurinic/apyrimidinic (AP) sites generated by HALFPIPE superfamily proteins are cleaved by their inherent AP lyase activities, other AP endonuclease activities or heat-promoted β-elimination. Although the HALFPIPE superfamily protein R.PabI, obtained from a hyperthermophilic archaea, *Pyrococcus abyssi*, shows weak AP lyase activity, HALFPIPE superfamily proteins in mesophiles, such as R.CcoLI from *Campylobacter coli* and R. HpyAXII from *Helicobacter pylori*, show significant AP lyase activities. To identify the structural basis for the AP lyase activity of R.CcoLI, we determined the structure of R.CcoLI by X-ray crystallography. The structure of R.CcoLI, obtained at 2.35-Å resolution, shows that a conserved lysine residue (Lys71), which is stabilized by a characteristic β-sheet structure of R.CcoLI, protrudes into the active site. The results of mutational assays indicate that Lys71 is important for the AP lyase activity of R.CcoLI. Our results help to elucidate the mechanism by which HALFPIPE superfamily proteins from mesophiles efficiently introduce double-strand breaks to specific sites on double-stranded DNA.

## Introduction

Restriction enzymes recognize and cleave specific DNA sequences that are not protected by cognate methyltransferases. Among the restriction enzymes, type II restriction enzymes, which recognize specific DNA sequences and cleave at or near sequences, are widely utilized in the field of biotechnology in such applications as genetic engineering and DNA fingerprinting^1^. Type II restriction enzymes are classified into five groups depending on their structural motifs^2^: the PD-(D/E)XK superfamily^3,4^, the HNH superfamily^5,6^, the PLD superfamily^7^, the GIY-YIG superfamily^8^, and the HALFPIPE superfamily^9^. R.PabI from *Pyrococcus abyssi* is a useful example of HALFPIPE superfamily restriction enzymes. Although most type II restriction enzymes cleave DNA by their endonuclease activities using Mg^2+^ ions, R.PabI, which recognizes the sequence 5′-GTAC-3′, cleaves double-stranded DNA (dsDNA) without the addition of divalent cations at high temperatures^10^. In contrast to the general restriction enzymes, R.PabI does not cleave a phosphodiester bond of DNA but cleaves an *N*-glycosidic bond in its recognition sequence, similar to DNA glycosylases^11^. The apurinic/apyrimidinic (AP) site generated by R.PabI is cleaved by its weak AP lyase activities, other AP endonuclease activities or heat-promoted β-elimination^11,12^. To date, we have determined the structures of R.PabI in the DNA-free state; the sequence-nonspecific DNA-binding state; the intermediate state, which is important for the indirect readout of the recognition sequence; and the product DNA-binding state^9,11,13,14^ (see Supplementary Fig. S1 online). R.PabI forms a homodimer and has a positively charged HALFPIPE structure at the dimer interface^9^. R.PabI recognizes the sequence 5′-GTAC-3′ in dsDNA using the HALFPIPE region. The 5′-GTAC-3′ site in dsDNA is unwound by the binding of R.PabI dimer, and three residues (Tyr68, His211 and Asp214) of R.PabI catalyze the hydrolysis of the *N*-glycosidic bond between adenine and deoxyribose in the recognition sequence (see Supplementary Fig. S1 online). These results indicate that the HALFPIPE superfamily restriction enzymes are not restriction endonucleases but are restriction DNA glycosylases^15^.

DNA glycosylases are enzymes that are utilized in the DNA repair pathways of organisms. In the base excision repair (BER) pathway, a damaged base is recognized by a DNA glycosylase, and an *N*-glycosidic bond of the damaged base is subsequently cleaved. A DNA molecule cleaved by a DNA glycosylase is recognized and repaired by downstream enzymes of the BER pathway^16,17^. For example, the uracil DNA glycosylase (uracil *N-*glycosylase, UNG) recognizes a uracil in dsDNA, which is generated by cytosine deamination or misincorporation of deoxyuridine monophosphate during replication^18,19^. The 8-oxoguanine DNA glycosylase (OGG1) recognizes an 8-oxoguanine in dsDNA, which is generated by guanine oxidation^20,21^. Among DNA glycosylases, some groups of DNA glycosylases, including OGG1, not only cleave *N*-glycosidic bonds but also cleave phosphodiester bonds through their inherent AP lyase activities (bifunctional DNA glycosylases). In contrast, other DNA glycosylases, including UNG, only hydrolyze *N*-glycosidic bonds (monofunctional DNA glycosylases). The AP lyase activities of bifunctional DNA glycosylases require an amine group, including in the N-terminal threonine^22^ or proline^23^ or in the side chain of lysine^24,25^; after the cleavage of an *N*-glycosidic bond and the generation of a reactive oxocarbenium intermediate, which are promoted by a catalytic acidic residue, an amine group forms an iminium crosslink with C1′ of the deoxynucleotide to cleave the DNA backbone through β- and δ-eliminations (see Supplementary Fig. S2 online)^26,27^.

**Figure 1:**
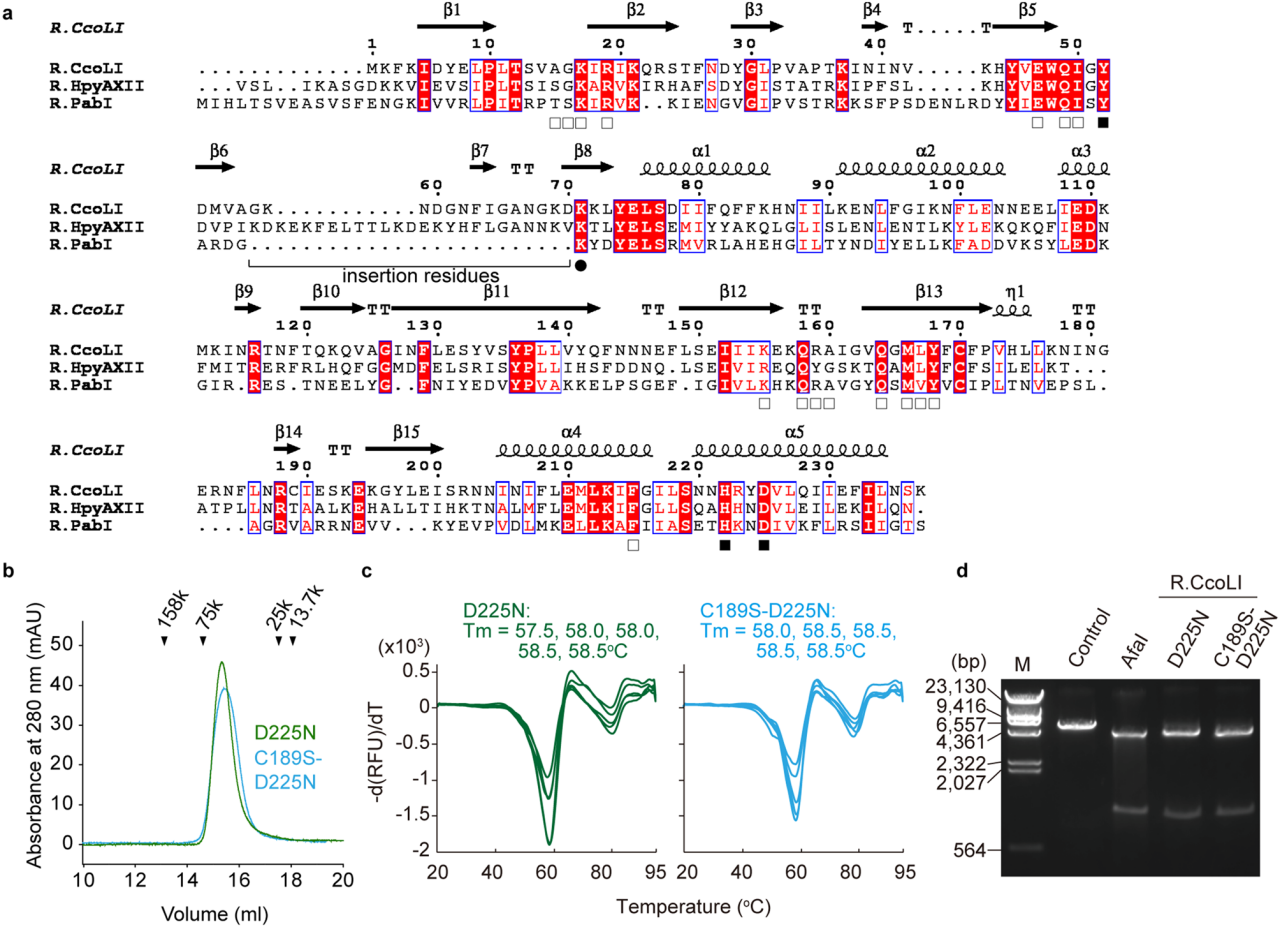
Preparation of R.CcoLI. (**a**) Sequence alignment of R.PabI homologs (R.CcoLI, WP_002830209; R. HpyAXII, ACI43084.1; R.PabI, CAB49082). Invariant residues are highlighted with red boxes, and conserved residues are shown in red text. The secondary structure of R.CcoLI (chain A) is indicated by helixes (α and 3_10_ (η)-helices), arrows (β-strand), and TT (β-turn). The catalytic triad and base recognition residues of R.PabI are marked with black and open squares, respectively. The residue that is predicted to be involved in the DNA lyase activity of R.CcoLI (Lys71) is marked with a circle. Insertion residues that are not conserved in R.PabI are indicated by a black line. (**b**) Gel filtration of the R.CcoLI(D225N) (*M*_r_ = 28,007) and R.CcoLI(C189S-D225N) (*M*_r_ = 27,991) mutants. The peak positions of the marker proteins are indicated by black triangles. Molecular weights of the R.CcoLI(D225N) and R.CcoLI(C189S-D225N) mutants were estimated to be 56 k and 53 k, respectively. Data are representative of three independent experiments. (**c**) Thermostability assay of R.CcoLI mutants. The melting curves of the R.CcoLI(D225N) mutant and the R.CcoLI(C189S-D225N) mutant are shown by green and cyan lines, respectively (n = 5). RUF, relative fluorescence unit. (**d**) DNA cleavage activities of the R.CcoLI(D225N) and R.CcoLI(C189S-D225N) mutants. The substrate DNA was treated with R.CcoLI mutants or AfaI, which cleaves the DNA duplex at the same site as R.CcoLI. The image is a cropped gel image. The full image is in Supplementary Fig. S7 online.

At medium temperature (40 °C), R.PabI does not cleave the DNA backbone but only hydrolyzes the *N*-glycosidic bond of adenine to produce an AP site^11^. R.PabI exhibits weak AP lyase activity at high temperatures^12^. These observations indicate that R.PabI acts as a monofunctional DNA glycosylase at medium temperature. In contrast, R.PabI homologs from mesophiles, such as R.CcoLI from *Campylobacter coli* and R.HpyAXII from *Helicobacter pylori*, show significant AP lyase activities at 37 °C; R.CcoLI and R.HpyAXII recognize the sequence 5′-GTAC-3′ in dsDNA, similar to R.PabI, and cleave the DNA backbone at the adenine site^15^. Because AP lyase activities of known DNA glycosylases require an amine group near the active sites, R.CcoLI and R. HpyAXII are predicted to possess amine groups at their active sites that are not conserved in the structure of R.PabI. The amino acid sequence alignment of R.PabI, R.CcoLI and R.HpyAXII shows that R.CcoLI and R. HpyAXII have characteristic insertion residues adjacent to the active site residue (Tyr68 of R.PabI) (Fig. 1a). Although this insertion may be related to the AP lyase activities of R.CcoLI and R. HpyAXII, the structure of the insertion residue has not been determined to date. To demonstrate the structural basis for the AP lyase activity of R.CcoLI, we determined the crystal structure of R.CcoLI at 2.35-Å resolution. The R.CcoLI structure and accompanying biochemical data showed that the characteristic insertion residues of R.CcoLI form an antiparallel β-sheet near the catalytic residues and that Lys71 in the antiparallel β-sheet is utilized for AP lyase activity. The HALFPIPE superfamily restriction enzymes exist both in mesophiles, such as *C. coli* and *H. pylori*, and in hyperthermophiles, such as *P. abyssi*. Comparing the structures and activities of R.CcoLI and R.PabI may help to elucidate the mechanism by which HALFPIPE superfamily restriction enzymes adapt to a wide range of temperature conditions.

## Results

### Determining the structure of R.CcoLI

To determine the crystal structure of R.CcoLI, we overexpressed the D225N mutant of R.CcoLI in *E. coli*. Asp225 of R.CcoLI corresponds to the active site residue of R.PabI (Asp214) (Fig. 1a). Asp214 of R.PabI is employed to stabilize the oxocarbenium ion intermediate, to deprotonate the catalytic water and to bind substrate DNA; the D214N mutation reduces the DNA glycosylase activity and the substrate DNA-binding ability of R.PabI^11^. Similar to the D214N mutation of R.PabI, the D225N mutation of R.CcoLI is predicted to reduce its DNA glycosylase activity. The D225N mutation was introduced to R.CcoLI to overexpress toxic proteins in *E. coli* cells. After Ni–NTA affinity chromatography, ion-exchange chromatography and gel-filtration chromatography purification, the highly purified R.CcoLI(D225N) mutant was obtained (see Supplementary Fig. S3 online). The gel-filtration analysis showed that R.CcoLI(D225N) forms a homodimer in solution similar to R.PabI (Fig. 1b). The melting temperature of the R.CcoLI(D225N) mutant was 57.5–58.5 °C (Fig. 1c). The R.CcoLI(D225N) mutant retained sequence-specific DNA cleavage activity (Fig. 1d and Supplementary Fig. S4 online). Although we could obtain crystals of the R.CcoLI(D225N) mutant and their X-ray diffraction data, the refinement statistics of the R.CcoLI(D225N) mutant structure were poor. To improve the quality of the crystals, the C189S mutation was introduced to the R.CcoLI(D225N) mutant. Cys189 of R.CcoLI corresponds to Val181 of R.PabI. The side chain of R.PabI Val181 is exposed to a solvent and does not interact with dsDNA^11,13,14^. The C189S mutation of R.CcoLI was predicted to reduce the heterogeneity of R.CcoLI that was caused by the oxidation of Cys189. The R.CcoLI(C189S-D225N) mutant was overexpressed and purified by a similar method to that employed for the R.CcoLI(C225N) mutant (Fig. 1b). The C189S mutation did not affect the melting temperature or the sequence-specific DNA cleavage activity of R.CcoLI (Fig. 1c,d). These results suggested that the C189S mutation does not affect the function of R.CcoLI.

**Figure 2:**
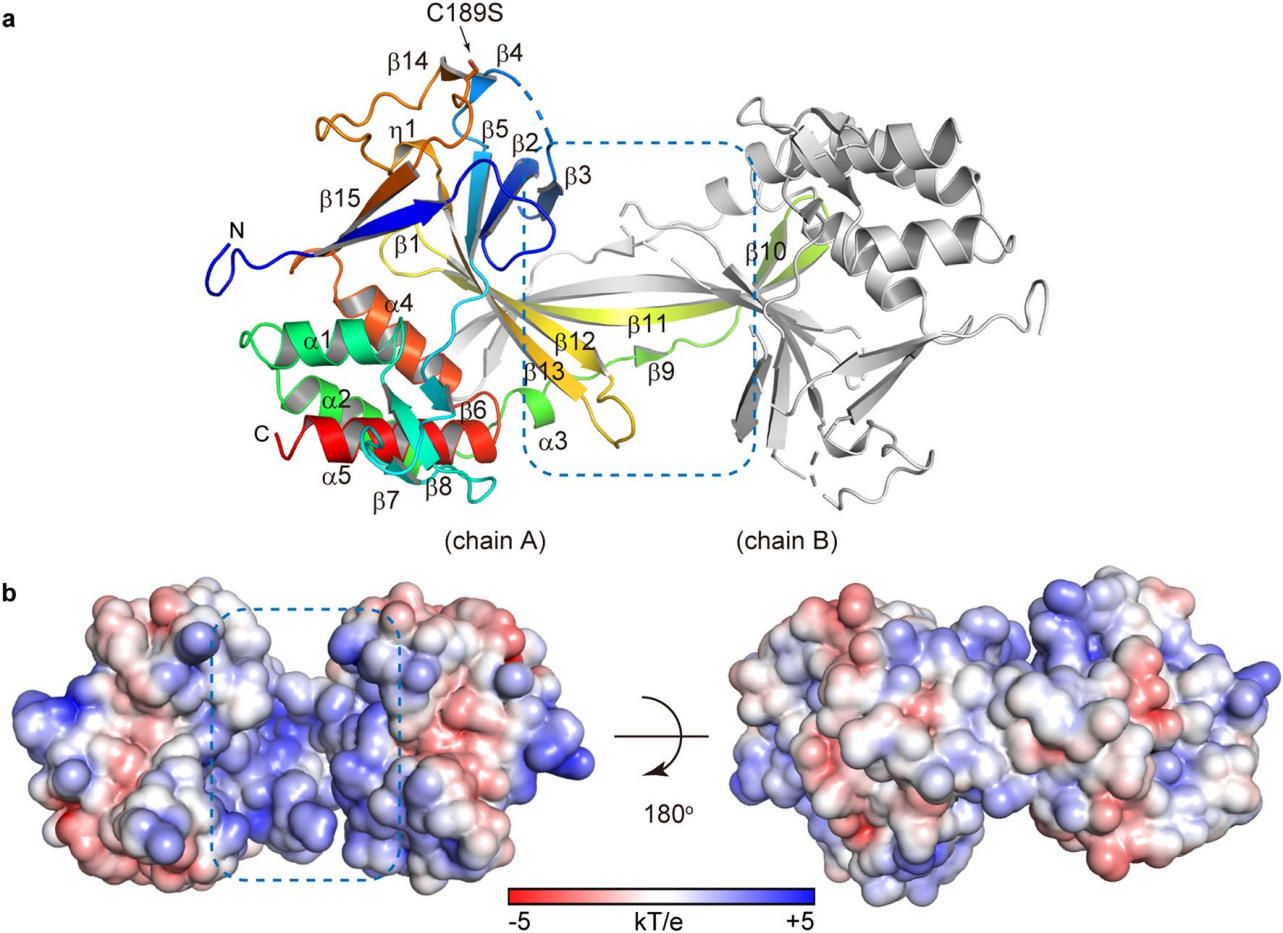
Overall structure of R.CcoLI. (**a**) Ribbon diagram of the R.CcoLI dimer. The ribbon of one R.CcoLI protomer (chain A) is colored blue (in the N terminus) to red (in the C terminus). The other R.CcoLI protomer (chain B) is colored gray. The HALFPIPE region of R.CcoLI dimer is indicated by a blue dotted square. The side chain of C189S is shown by a stick model. (**b**) The ± 5 kT/e electrostatic potential of the R.CcoLI dimer is plotted on the solvent-accessible surface.

The R.CcoLI structure was determined at a resolution of 2.35 Å using the crystal of the R.CcoLI(C189S-D225N) mutant. The final model contained one R.CcoLI dimer (chains A and B) in the asymmetric unit (Fig. 2a and Supplementary Fig. S5 online). R.CcoLI consists of five α-helices, fifteen β-strands and one 3_10_ (η)-helix and forms the characteristic HALFPIPE structure using strands β3-β2-β5-β13-β12-β11. Similar to the R.PabI structure, the HALFPIPE region of R.CcoLI has a positively charged surface (Fig. 2b)^9,11,13,14^. Due to poor electron density, the structure models of the β2-β3 and β3-β4 loops of chain A, the β1–β2 loop, the β2–β5 region, the β6–β7 loop, the β11–β12 loop, the β12–β13 loop and the η1-β15 region of chain B are not included in the final model. The final model contains only ten water molecules, despite the relatively high-resolution structure (2.35 Å). These properties cause the structure to exhibit a relatively high *R*_free_ value (Table 1). In the R.CcoLI structure, C189S is exposed to solvent, as predicted (Fig. 2a).

**Table 1:** Summary of data collection and refinement statistics of R.CcoLI.

**Data collection**	
Space group	*P*22_1_2_1_
**Cell dimensions**	
*a*, *b*, *c* (Å)	66.49, 89.60, 100.66
Resolution (Å)	47.17–2.35 (2.44–2.35)*
*R*_sym_ (%)	4.7 (79.2)
Mean (*I*/σ*I*)	23.1 (2.1)
Completeness (%)	99.9 (99.7)
Redundancy	7.2 (6.6)
CC (1/2)	0.999 (0.705)
**Refinement**	
*R*/*R*_free_ (%)	22.9/25.9
**No. atoms**	
R.CcoLI	3545
Water	10
***B-factors (Å***^**2**^**)**	
R.CcoLI	72.10
Water	59.04
**R.m.s deviations**	
Bond lengths (Å)	0.002
Bond angles (^o^)	0.384
**Ramachandran plot**	
Favored region (%)	98.0
Allowed region (%)	2.0

*The numbers in parentheses represent data for the highest-resolution shells.

### Structure comparison with R.PabI

**Figure 3:**
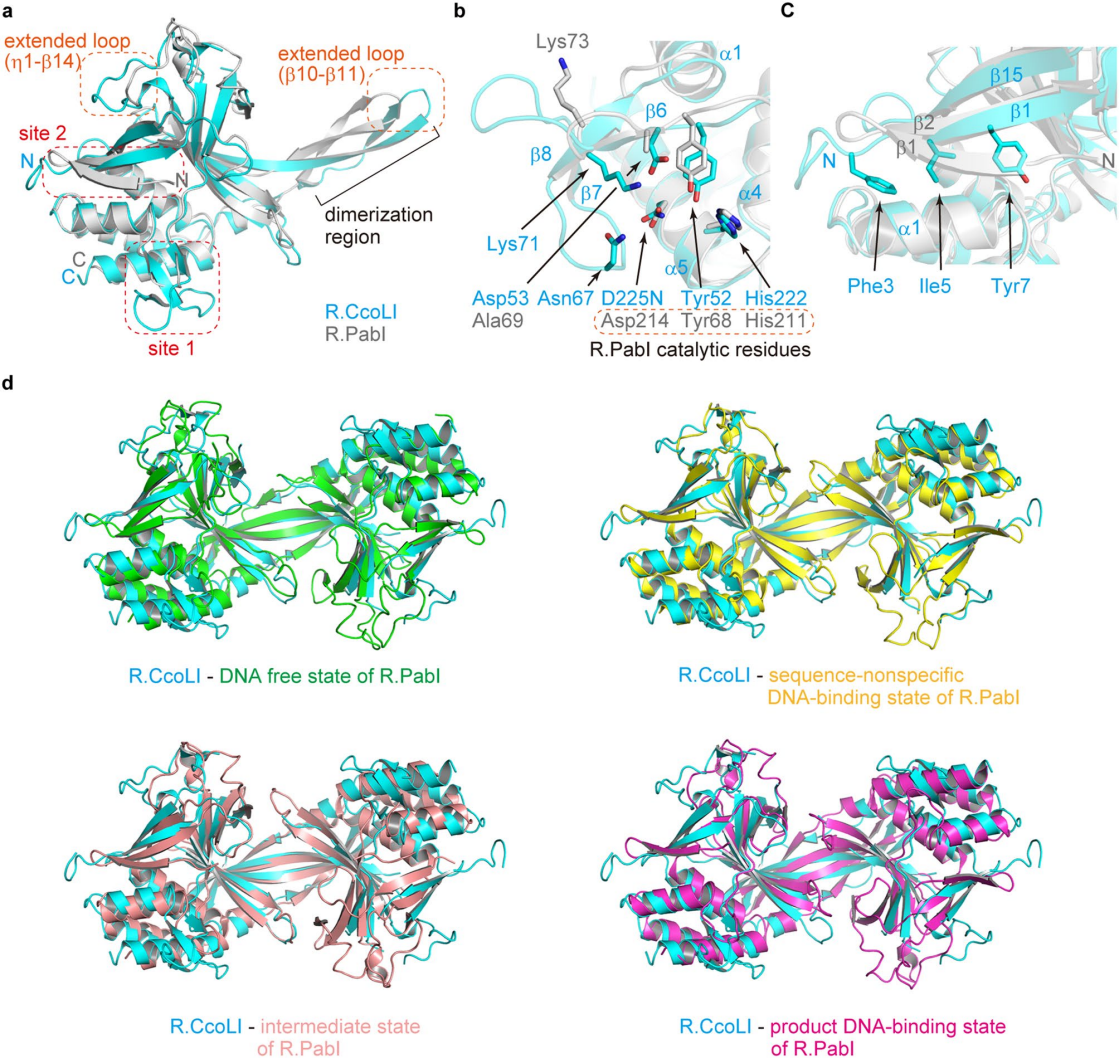
Comparison with R.PabI. (**a**) Characteristic structure of R.CcoLI. The structures of R.CcoLI protomer (chain A) and R.PabI protomer in the product DNA-binding state (PDB ID: 3WAZ, chain A) are superposed and are colored cyan and gray, respectively. Characteristic local structures in R.CcoLI that are not observed in R.PabI are indicated by red dotted squares (sites 1 and 2). The extended loops of R.CcoLI are indicated by orange dotted squares. (**b**) Structure of the insertion residues of R.CcoLI (site 1 in (A)). Residue numbers and secondary structures of R.CcoLI are labeled cyan. Residue numbers of R.PabI are labeled gray. (**c**) Structure of the N-terminal region of R.CcoLI (site 2 in (A)). Secondary structures of R.CcoLI and R.PabI are labeled cyan and gray, respectively. (**d**) Superposition of the dimeric structure of R.CcoLI (cyan) and those of R.PabI in the DNA-free state (PDB ID: 2DVY, green), the sequence-nonspecific DNA-binding state (5IFF, yellow), the intermediate state (PDB ID: 6L2O, salmon pink) and the product DNA-binding state (3WAZ, magenta).

When the structures of R.CcoLI and R.PabI (the product DNA-binding state, PDB: 3WAZ) were compared, the secondary structures of two regions (sites 1 and 2) were observed to be different between the proteins (Fig. 3a)^11^. Site 1 exists on helices α1, α4 and α5 of R.CcoLI. In this region of R.CcoLI, three catalytic residues of R.PabI, that is, Tyr68, His211 and Asp214, are conserved as Tyr52, His222 and Asp225, respectively. In the R.CcoLI structure, the insertion residues of R.CcoLI (Fig. 1a) forms an antiparallel β-sheet (β6-β8-β7) adjacent to the active site (Fig. 3b and Supplementary Fig. S5 online). According to this structure, the side chains of Asp53, Asn67 and Lys71 face the active site of R.CcoLI. Lys71 of R.CcoLI is conserved in R.PabI as Lys73 (Fig. 1a). However, in the R.CcoLI structure, the side-chain orientation of Lys71 is flipped to the active site due to the insertion of the R.CcoLI specific antiparallel β-sheet. In contrast, Asp53 and Asn67 are R.CcoLI-specific residues. Site 2 is the N-terminal region of R.CcoLI. In the structure of R.PabI, the N-terminal region forms a three-stranded antiparallel β-sheet. However, R.CcoLI lacks the first β-strand due to the shortage of the N-terminal residues (Fig. 1a) and forms a two-stranded antiparallel β-sheet (β1–β15, Fig. 3c). Because R.CcoLI lacks the first β-strand, the side chains of Phe3, Ile5 and Tyr7 are partially exposed to the solvent. In addition to these two sites, the lengths of two loops (β10–β11 and η1-β14 loops in R.CcoLI) also differ between R.CcoLI and R.PabI. In the R.PabI structure, the lengths of the β10–β11 and η1-β14 loops are shortened by two and five residues, respectively.

The structural comparison between R.CcoLI and R.PabI (the product DNA-binding state) shows that the structures of the dimerization region are also different (Fig. 3a). The structure of the R.PabI dimer is modified depending on the DNA binding state^9,11,13,14^ (see Supplementary Fig. S1 online). When the protomer structure of R.CcoLI (chain A) was compared to those of R.PabI in the DNA-free state (PDB: 2DVY), the sequence-nonspecific DNA-binding state (PDB: 5IFF), the intermediate state (PDB: 6L2O) and the product DNA-binding state (PDB: 3WAZ), the R.PabI structure in the sequence-nonspecific DNA-binding state showed the highest structural similarity (*Z*-score from the Dali server = 21.2, root-mean-square deviation (RMSD) = 1.9 Å, sequence identity = 26%)^28^, although the R.CcoLI structure was determined without DNA. The dimeric structure of R.CcoLI was also well superposed with the dimeric structure of R.PabI in the sequence-nonspecific DNA-binding state, and the RMSD was 2.3 Å for 298 superposed Cα atoms (Fig. 3d and Supplementary Fig. S6 online).

### Active site structure

**Figure 4:**
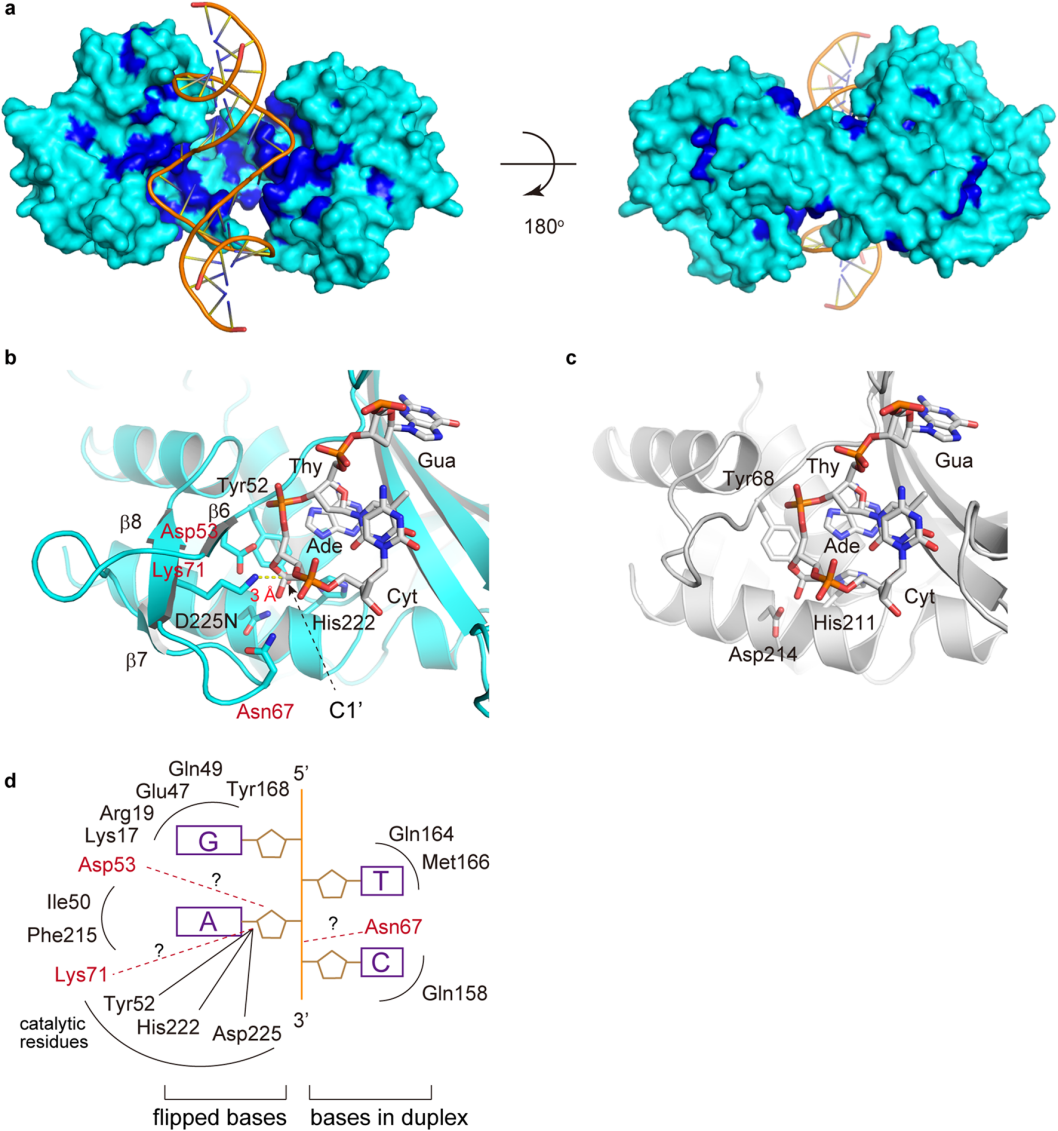
Active site structure. (**a**) R.CcoLI-DNA binding model. The model was prepared using the coordinates of R.CcoLI and the R.PabI-product DNA complex (PDB ID: 3WAZ). The sequence conservation of Fig. 1A is superposed on the molecular surface of R.CcoLI. The molecular surface of invariant residues is colored blue. The molecular surface of the other residues is colored cyan. The DNA model is shown as an orange cartoon. (**b**) DNA binding model of R.CcoLI around the active site. R.CcoLI is colored cyan. The DNA model of the sequence 5′-GTAC-3′ is shown using a white stick model. The active site residues and residues near the catalytic site are shown by stick models. Residues in the characteristic antiparallel β-sheet region of R.CcoLI are labeled red. (**c**) Product DNA-binding structure of R.PabI around the active site. R.PabI is colored gray. (**d**) Plausible DNA recognition mechanism of R.CcoLI. The DNA backbone is shown as an orange line. R.CcoLI residues that are conserved in R.PabI are colored black. R.CcoLI-specific residues are colored red. DNA bases are colored purple.

The amino acid sequence alignment of R.PabI homologs shows that most of the conserved residues are located near the positively charged HALFPIPE region (Figs. 1a, 2b and 4a). R.CcoLI is predicted to recognize a negatively charged dsDNA in the HALFPIPE region, similar to R.PabI. To elucidate the DNA cleavage mechanism of R.CcoLI, we created the R.CcoLI-product DNA complex model using the coordinates of R.CcoLI and that of R.PabI in the product DNA binding state (PDB: 3WAZ) (Fig. 4a,b)^11^. The model structure of the R.CcoLI-product DNA complex shows that the catalytic residues of R.CcoLI (Tyr52, His222 and Asp225) clearly exist near the cleaved *N*-glycosidic bond of the adenine in the recognition sequence (Fig. 4b,c). Among the base-recognizing residues of R.PabI, ten residues are conserved in R.CcoLI (Fig. 1a). According to the amino acid sequence similarity, Lys17, Arg19, Glu47, Gln49 and Tyr168 of R.CcoLI are predicted to recognize a guanine base in the recognition sequence; Gln164 and Met166 of R.CcoLI are predicted to recognize a thymine base in the recognition sequence; Ile50 and Phe215 of R.CcoLI are predicted to recognize an adenine base in the recognition sequence, and Gln158 is predicted to recognize a cytosine base in the recognition sequence (Fig. 4d). In addition to these residues, Asp53, Asn67 and Lys71 of R.CcoLI are located near the active site due to the insertion of the characteristic antiparallel β-sheet (Fig. 4b,c). The model structure of the R.CcoLI-product DNA complex shows that the distance between the side-chain amine group of Lys71 and the C1′ carbon atom of cleaved deoxyadenosine is approximately 3 Å (Fig. 4b). Because the AP lyase activity of DNA glycosylase is initiated by iminium crosslink formation between C1′ and an amine group^22–27^ (see Supplementary Fig. S2 online), Lys71 of R.CcoLI is predicted to be an important residue for the AP lyase activity of R.CcoLI. In contrast, the side chains of Asp53 and Asn67 are located near the O4′ oxygen atom of deoxyribose and the phosphate group of the DNA backbone, respectively (Fig. 4b,d). These resides are predicted to be utilized for the stabilization of the R.CcoLI-DNA complex.

### Mutation assay

**Figure 5:**
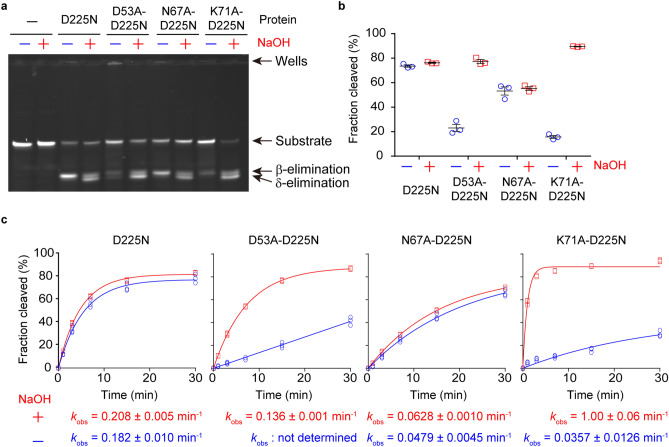
Mutation assay. (**a**) DNA glycosylase activity assay of the R.CcoLI mutants at 37 °C for 15 min. After the reaction, half of the solution was treated with NaOH to generate strand breaks at AP sites (β- and δ-eliminations). Data are representative of three independent experiments. The image is a cropped gel image. The full image is in Supplementary Fig. S7 online. Fraction cleaved = volume of cleaved DNA/(volume of cleaved DNA + volume of uncleaved DNA + volume of DNA stuck in the well) (**b**) Quantification of **a**. The fractions of products are indicated as blue circles and red squares. Data are the means ± SEM from three independent experiments. (**c**) Kinetics experiments for the DNA glycosylase activities of the R.CcoLI mutants at 37 °C. The enzymatic rate constant and its standard error were obtained from a single-exponential fit to the data from three independent measurements.

The model structure of the R.CcoLI-DNA complex suggests that Asp53, Asn67 and Lys71 are important for the catalytic activity of R.CcoLI; in particular, the side-chain amine group of Lys71 is predicted to be important for the AP lyase activity of R.CcoLI. To analyze the importance of the side-chain atoms of these residues, we prepared the D53A-D225N, N67A-D225N and K71A-D225N mutants and analyzed their DNA glycosylase activities (Fig. 5a–c). In this study, we analyzed the effects of mutations using the D225N mutant as a control. Asp225 of R.CcoLI is predicted to be used to cleave the *N*-glycosidic bond and to generate the oxocarbenium intermediate. The D225N mutation was predicted to reduce the efficiency of this reaction, although the D225N mutant exhibited sequence-specific DNA cleavage activity (Fig. 1d). If the AP lyase activity of R.CcoLI is mediated by the interaction between the side-chain amine group of Lys71 and DNA, the K71A mutant of R.CcoLI acts as a monofunctional DNA glycosylase that hydrolyzes the *N*-glycosidic bond; the oxocarbenium intermediate is not attacked by the amine group of Lys71 but is attacked by water to generate an AP site. The DNA backbone of an AP site is unstable and is easily cleaved by NaOH treatment at the 3′ and 5′ sides of the AP sites (β- and δ-eliminations, respectively)^11,15^. The results of the DNA glycosylase assay of the R.CcoLI(D225N) mutant showed that the fractions of cleaved DNA were the same, regardless of whether NaOH was added. This finding indicated that the D225N mutant of R.CcoLI functions as a bifunctional DNA glycosylase. In contrast, the results of the DNA glycosylase assay of the K71A-D225N mutant showed that the fraction of cleaved DNA was highly increased by NaOH treatment. This result indicated that the K71A-D225N mutant of R.CcoLI functions as a monofunctional DNA glycosylase and that the side-chain amine group of Lys71 is important for the DNA cleavage activity of R.CcoLI. The reaction speed of the K71A-D225N mutant was determined to be higher than that of the D225N mutant (Fig. 5c). This finding suggested that the Lys71-dependent β-elimination is the rate-limiting step of the R.CcoLI activity. Notably, the K71A-D225N mutant slightly cleaved the substrate DNA in the absence of NaOH treatment (Fig. 5a–c); the K71A-D225N mutant also functioned as a bifunctional DNA glycosylase.

Although Asp53 does not possess amine groups, the results of the DNA glycosylase assay of the D53A-D225N mutant also showed a cleavage pattern similar to that of the K71A-D225N mutant; the fraction of cleaved DNA was highly increased by NaOH treatment (Fig. 5a–c). Because the side chain of Asp53 is located adjacent to the side chain of Lys71 and the O4′ oxygen atom of deoxyribose (Fig. 4b), the D53A mutation might decrease the stability of the side chain of Lys71 and deoxyribose, while the increased flexibility in the D53A-D225N mutant is predicted to reduce the efficiency of iminium crosslink formation between Lys71 and DNA. The results of the DNA glycosylase assay examining the N67A-D225N mutant showed that the cleavage activity was reduced compared to that of the D225N mutant, regardless of whether NaOH was added (Fig. 5a–c). This result suggested that the side chain of Asn67 is not employed for DNA backbone cleavage; also, Asn67 is predicted to be used for DNA stabilization (Fig. 4b,d).

## Discussion

The HALFPIPE superfamily protein of restriction enzymes was first discovered in the hyperthermophilic archaea *P. abyssi* and was designated R.PabI. Structural studies have identified that HALFPIPE superfamily proteins are not restriction endonucleases, but rather are restriction DNA glycosylases. Because hyperthermophiles, including *P. abyssi,* grow optimally at high temperatures (over 80 °C), proteins from these organisms possess extremely high thermal stability; in fact, R.PabI cleaves dsDNA at temperatures ranging from 60 to 90 °C ^10^. Mesophiles, such as *Campylobacter* and *Helicobacter*, also have HALFPIPE superfamily proteins. Although R.PabI is still active above 80 °C, R.CcoLI from *C. coli* is denatured at temperatures above approximately 60 °C (Fig. 1c). The most striking difference between R.PabI and R.CcoLI is that R.CcoLI cleaves the DNA backbone at medium temperatures, while the DNA backbone cleavage activity of R.PabI is very low at medium temperatures; R.PabI only hydrolyzes the *N*-glycosidic bond of the recognition sequence at medium temperatures^11^. Both R.PabI and R.CcoLI are members of the HALFPIPE superfamily of restriction enzymes. However, at medium temperatures, R.PabI acts as a monofunctional DNA glycosylase, while R.CcoLI acts as a bifunctional DNA glycosylase. To demonstrate the structural basis for the functional switching of these proteins, that is, the switching between the monofunctional DNA glycosylase and the bifunctional DNA glycosylase, we determined the crystal structure of the R.CcoLI(C189S-D225N) mutant. The most striking feature of the R.CcoLI structure is that the insertion residues that are not conserved in R.PabI form the characteristic antiparallel β-sheet structure (β6, β7 and β8) (Fig. 3a,b). Due to the formation of the antiparallel β-sheet structure, the side-chain amine group of Lys71 is located near the *N*-glycosidic bond of deoxyadenosine (Fig. 4b). Lys71 of R.CcoLI is conserved in R.PabI as Lys73. The superposition of the structures of R.CcoLI and R.PabI shows that the Cα atom of R.CcoLI Lys71 is close to that of R.PabI Lys73 (~ 3 Å). However, the R.CcoLI-specific β-sheet formation causes the inversion of the side chain direction of Lys71 (Fig. 3b). The enzymatic activity assays demonstrated that Lys71 of R.CcoLI is important for AP lyase activity (Fig. 5a–c). These results indicate that the AP lyase activity of R.CcoLI is facilitated by the insertion of the antiparallel β-sheet structure near the active site. The amino acid sequence of the characteristic antiparallel β-sheet structure of R.CcoLI is largely conserved in R.HpyAXII (Fig. 1a), which is the R.PabI homolog from mesophiles that also shows significant AP lyase activity^15^. Although the structure of R. HpyAXII has not been determined, this sequence similarity suggests that the corresponding region of R. HpyAXII forms an antiparallel β-sheet structure similar to R.CcoLI. The insertion of the antiparallel β-sheet structure in this region is predicted to be a signature of the HALFPIPE superfamily enzymes with AP lyase activity (bifunctional DNA glycosylase). As mentioned in the results section, the K71A-D225N mutant showed weak DNA cleavage activity. R.CcoLI is also predicted to cleave dsDNA through a Lys71-independent mechanism. However, the Lys71-independent DNA cleavage mechanism of R.CcoLI has not been elucidated to date.

**Figure 6:**
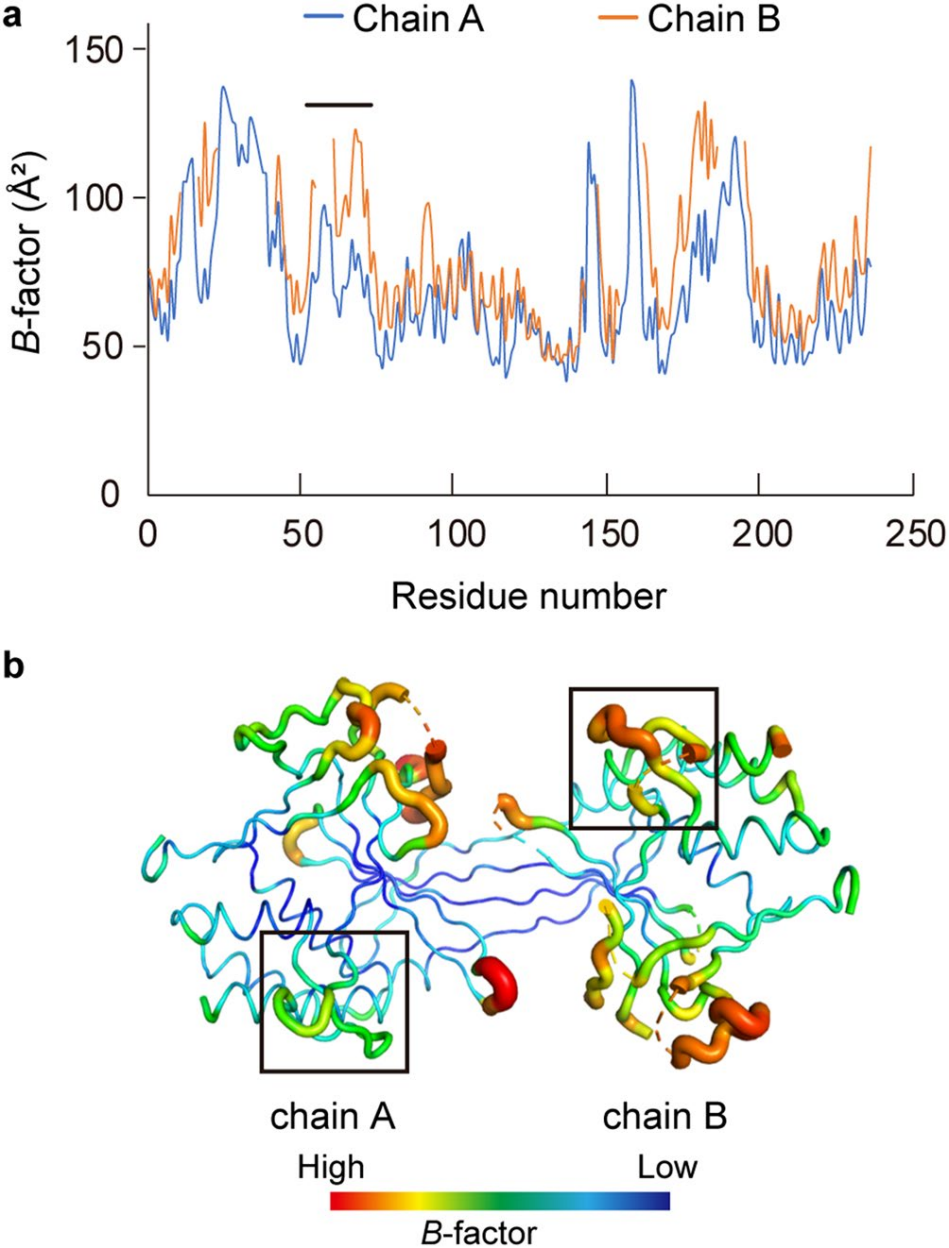
Temperature factor of R.CcoLI. (**a**) Plot of the average *B*-factor of each residue. The position of the characteristic antiparallel β-sheet region is indicated by a black line. (**b**) The R.CcoLI structure is colored according to the *B*-factors. The positions of the characteristic antiparallel β-sheet regions are indicated by black boxes.

In general, the thermostabilities of proteins have been attributed to several factors: increased numbers of ion-pair networks on protein surfaces, loop shortening and decreased numbers of hydrophobic accessible surface areas^29–33^. In the R.PabI structure, loop regions that correspond to the β10–β11 and η1–β14 loops of R.CcoLI are shortened compared to the structure of R.CcoLI (Fig. 3a). These loop shortenings are predicted to be important for the high thermostability of R.PabI. The existence of the additional antiparallel β-sheet structure near the active site is characteristic of R.CcoLI. However, this structure is truncated in R.PabI (Fig. 3a,b). In the R.CcoLI structure, this region shows relatively high temperature factors compared to the protein core region (Fig. 6a,b and Supplementary Fig. S5 online). It is predicted that the truncation of this region is also important for the high thermostability of R.PabI. In the R.CcoLI structure, the hydrophobic residues in the N-terminal region (Phe3, Ile5 and Tyr7) are exposed to the solvent. In contrast, the corresponding region of R.PabI is covered by the additional β-strand (Fig. 3c). Therefore, the hydrophobic surface area of this region is decreased in the R.PabI structure. It is predicted that this difference contributes to the high thermostability of R.PabI. The AP lyase activity of R.CcoLI is mediated by Lys71 in the characteristic antiparallel β-sheet structure. In contrast, R.PabI only shows weak AP lyase activity due to the lack of a lysine amine group near the active site (Fig. 3b). Because *C. coli* is a mesophile, high thermostability is not necessary for R.CcoLI; R.CcoLI can utilize the relatively flexible regions (that is, the antiparallel β-sheet structure containing Lys71) for its catalytic mechanism. Meanwhile, R.PabI from the hyperthermophile *P. abyssi* must possess high thermostability to function at high temperature. Because AP sites are unstable in high-temperature conditions^11^, AP lyase activity is predicted not to be required for the DNA damaging function of R.PabI. R.PabI might have relinquished its AP lyase activity to obtain high thermostability.

The structures of restriction enzymes are frequently modified by the binding of DNA. For example, the structures of such restriction endonucleases as EcoRV and BamHI, which belong to the PD-(D/E)XK superfamily, show that these proteins widen their DNA binding clefts when they weakly bind sequence-nonspecific dsDNA, and the clefts become narrow when they tightly bind their recognition sequences in dsDNA; the weak sequence-nonspecific dsDNA-binding states are utilized for facilitated diffusions on dsDNA^3,4,34^. Our previous studies also demonstrated that the structure of R.PabI is modified by the binding of dsDNA^11,13,14^. Although the structure of R.CcoLI was determined in the absence of DNA, the R.CcoLI structure is most similar to the R.PabI structure in the sequence-nonspecific dsDNA-binding state (Fig. 3d and Supplementary Fig. S6 online). This structural similarity might indicate that the R.CcoLI structure is not modified by the binding of sequence-nonspecific dsDNA. However, the precise DNA recognition mechanism of R.CcoLI will be clarified by the determination of the R.CcoLI-dsDNA complex structure. In this study, we analyzed the structure and function of R.CcoLI using the D225N mutant. Structural and functional studies utilizing the wild-type enzyme may demonstrate the function of R.CcoLI more precisely.

## Methods

### Expression and purification

The gene fragment of R.CcoLI (NCBI Reference Sequence: WP_002830209) was synthesized by GenScript (see Supplementary Table S1 online). Each codon in the synthesized gene was optimized for expression in *E. coli*. The R.CcoLI gene fragment was amplified by PCR using primers in Supplementary Table S2 online (Cloning-F, R), and it was cloned into the SmaI-HindIII site of pET48b (pET48b-R.CcoLI) to express R.CcoLI with an N-terminal thioredoxin tag. To reduce the cytotoxicity of R.CcoLI, the D225N mutation, which corresponds to the D214N mutation of R.PabI (Fig. 1a), was introduced to pET48b-R.CcoLI plasmid using the PrimeSTAR Mutagenesis Basal Kit (TAKARA) and primers (D225N-F, R) in Supplementary Table S2 online (pET48b-R.CcoLI(D225N)). For R.CcoLI expression, the pET48b-R.CcoLI(D225N) plasmid was transformed into *E. coli* Rosetta (DE3) pLysS (Novagen). The recombinant *E. coli* cells were cultivated at 37 °C in LB medium supplemented with 20 μg/ml kanamycin and 50 μg/ml chloramphenicol until the optical density of the medium at 600 nm reached 0.8. The expression of R.CcoLI(D225N) was induced by the addition of isopropyl β-d-1-thiogalactopyranoside (IPTG) at a final concentration of 0.1 mM. After overnight cultivation at 18 °C, the cells were harvested by centrifugation at 5000 × *g* for 10 min at 4 °C.

The harvested cells were resuspended in 50 mM Tris HCl pH 8.0, 10 mM imidazole and 1 mM Tris(2-carboxyethyl)phosphine (TCEP) and lysed by sonication. After centrifugation at 40,000 × g for 30 min at 4 °C, the supernatant was mixed with 2 ml of Ni–NTA Superflow resin (QIAGEN). The resin was washed using 20 ml of 50 mM Tris HCl pH 8.0, 20 mM imidazole and 1 mM TCEP, and the bound protein was eluted using 10 ml of 50 mM MES pH 6.0, 200 mM imidazole, 50 mM MgCl_2_ and 1 mM TCEP. The eluted protein was treated with HRV3C protease at 4 °C overnight to remove the N-terminal thioredoxin tag. The protein that the expression tag had been removed from was purified using a Mono S HR 10/10 (GE Healthcare) column pre-equilibrated with 10 mM MES pH 6.0, 200 mM NaCl and 1 mM TCEP, and the protein was eluted with a linear gradient of 0.2–1.5 M NaCl. The protein was further purified using a Superdex 200 h 10/30 (GE Healthcare) column pre-equilibrated with 10 mM MES pH 6.0, 200 mM NaCl and 1 mM TCEP. The protein sample was stored at − 80 °C until use.

Expression vectors of R.CcoLI(D225N) mutants (the D53A-D225N, N67A-D225N, K71A-D225N and C189S-D225N mutants) were prepared using the PrimeSTAR Mutagenesis Basal Kit (TAKARA) and primers in Supplementary Table S2 online. Each mutant was expressed and purified using the same method as that described above.

### Oligomeric state analysis by gel filtration chromatography

The samples (2.3 μM) were loaded onto a Superdex 200 h 10/30 (GE Healthcare) column and eluted with buffer containing 10 mM MES pH 6.0, 200 mM NaCl and 1 mM TCEP. To estimate the oligomeric state of R.CcoLI, the following standard proteins were used: aldolase (*M*_r_ = 158,000), conalbumin (*M*_r_ = 75,000), chymotrypsinogen A (*M*_r_ = 25,000), and ribonuclease A (*M*_r_ = 13,700).

### DNA cleavage assay

A modified pET26b plasmid, possessing only one 5′-GTAC-3′ site, was employed as a substrate for R.CcoLI (see Supplementary Fig. S4 online)^11^. The modified pET26b plasmid was cut with HindIII (TAKARA) to linearize the plasmid. To analyze the DNA cleavage activity of R.CcoLI, 0.2 μg of R.CcoLI mutants and 0.2 μg of the linearized plasmid were mixed in 0.1 M sodium phosphate buffer pH 6.5 and 1 mM TCEP and were incubated at 37 °C for 30 min. The cleaved DNAs were separated by electrophoresis through a 1% agarose gel. The DNAs were visualized with blue-LED light after GelGreen (Biotium) staining. Products by AfaI (TAKARA), which cleaves the sequence 5′-GTAC-3′, were also separated as a control.

### DNA glycosylase activity assay

DNA glycosylase activity assays of R.CcoLI mutants were performed using 24-bp dsDNA containing one 5′-GTAC-3′ sequence (5′-fluorecesin-GGATGCATGAGTACGAGGACCATC-3′, see Supplementary Fig. S4 online). A total of 0.2 μM of the substrate dsDNA was mixed with 0.8 μM of the R.CcoLI dimer in a reaction buffer (0.1 M sodium phosphate buffer pH 6.5, 1 mM TCEP). The reaction solutions were incubated at 37 °C for 15 min or for 1, 3, 7, 15 and 30 min. After the enzymatic reaction, the solutions were supplemented with 0.1 M NaOH or 0.1 M HCl to terminate the enzymatic reaction. To cleave the 5′ and 3′ sides of the AP sites generated by R.CcoLI, the solutions supplemented with NaOH were heated at 70 °C for 10 min. The reaction solutions were neutralized by the addition of an equal concentration of HCl or NaOH and separated on a denaturing 18% polyacrylamide gel in 0.5 × TBE and 7 M urea. The fluorescence was measured using an Amersham Imager 680 (GE Healthcare). Data were quantified using the program Amersham Imager 680 Analysis Software (GE Healthcare). The enzymatic rate constant *k*_obs_ was obtained from a single-exponential fit to the data from three independent measurements: *f*_p_ = *f*_p_max × (1 − e^−*kt*^), where *f*_p_ is the fraction of product, *f*_p_max is the maximum value of *f*_p_, and *t* is the time of the reaction (min).

### Denaturation assay

For the denaturation assay of R.CcoLI, proteins (10 μM) and 2.5 × SYPRO Orange (Thermo Fisher Scientific) were mixed in 0.1 M sodium phosphate buffer pH 6.5 and 1 mM TCEP. Denaturation assays were performed using a CFX Connect Real-Time PCR Detection System (Bio-Rad Laboratories). Fluorescence was measured from 20 to 95 °C in 0.5 °C steps (excitation, 450–490 nm; detection, 560–580 nm). Data were analyzed using Bio-Rad CFX Manager 3.0 software.

### Crystallization, data collection and structure determination

The purified protein was concentrated to 18 mg/ml using Vivaspin 6 (MWCO 30 k Da, Sartorius) for crystallization. Crystallization experiments of the R.CcoLI(C189S-D225N) mutant were performed using the sitting-drop vapor-diffusion method at 20 °C. The crystals of R.CcoLI(C189S-D225N) were obtained using a reservoir solution of 0.1 M MES pH 6.0 and 8% PEG6000 one day later. X-ray diffraction data of the R.CcoLI(C189S-D225N) crystal were collected on the AR-NW12A beamline at the Photon Factory (Tsukuba, Japan) under cryogenic conditions (95 K). For cryoprotection, the R.CcoLI(C189S-D225N) crystal was soaked in a reservoir solution supplemented with 40% ethylene glycol for several seconds. The R.CcoLI(C189S-D225N) crystal diffracted X-rays to 2.35-Å resolution. The X-ray diffraction data were indexed, integrated and scaled with XDS^35^. The R.CcoLI(C189S-D225N) crystal belonged to the space group *P*22_1_2_1_ with unit cell parameters of *a* = 66.49 Å, *b* = 89.60 Å and *c* = 100.66 Å. The crystal contains two R.CcoLI(C189S-D225N) molecules per asymmetric unit according to the Matthews coefficient (*V*_M_ = 2.70 Å^3^Da^−1^)^36^.

The initial model of R.CcoLI(C189S-D225N) was determined by the molecular replacement method using the program Phaser^37^. The ensemble of the R.PabI structures (the DNA-free state (PDB code: 2DVY)^9^, the product DNA-binding state (PDB code: 3WAZ)^11^ and the sequence-nonspecific DNA-binding state (PDB code: 5IFF)^13^) was used as the search model. The initial model was refined and rebuilt using the programs Phenix.refine^38^ and Coot^39^. The final model of R.CcoLI(C189S-D225N) was refined to 2.35 Å resolution with *R* and *R*_free_ values of 22.9% and 25.9%, respectively. The geometry of the final model was evaluated using the program MolProbity^40^. In the Ramachandran plot, 98.0% of the residues were in the favored region, and the rest were in the allowed region. The data collection and refinement statistics are summarized in Table 1.

### Computational analysis

The protein structure was analyzed using a set of computer programs as follows: PISA^41^ for the analysis of the protein interface, surface and assemblies, APBS^42^ for the calculation of macromolecular electrostatics, Dali for the search for similar structures from the database^28^, Clustal Omega^43^ for the amino acid sequence alignment, ESpript^44^ for the preparation of alignment figure, and PyMOL (http://pymol.org) for the depiction of structures.

## Supplementary Information

Supplementary Information.

## Data Availability

Atomic coordinates and structure factors for the reported crystal structures have been deposited with the Protein Data Bank under accession number 7CFA.
