# Supplementary material for: Crystal structure and DNA cleavage mechanism of the restriction DNA glycosylase R.CcoLI from Campylobacter coli
Source: Sci Rep. 2021 Jan 13;11:859. doi: 10.1038/s41598-020-79537-y (PMC7806768; doi:10.1038/s41598-020-79537-y)
Supplement: Supplementary file 1 — Supplementary Information. [file 41598_2020_79537_MOESM1_ESM.pdf]

Supplementary Information

Crystal structure and DNA cleavage mechanism of the restriction DNA glycosylase R.CcoLI from *Campylobacter coli*

Ken-ichi Miyazono, Delong Wang, Tomoko Ito, and Masaru Tanokura*

Department of Applied Biological Chemistry, Graduate School of Agricultural and Life Sciences, The University of Tokyo, 1-1-1 Yayoi Bunkyo-ku, Tokyo, 113-8657, Japan

*Corresponding author: Masaru Tanokura

Phone: +81-3-5841-5165

Fax: +81-3-5841-8023

E-mail: amtanok@mail.ecc.u-tokyo.ac.jp

Supplementary Table S1. Synthesized R.CcoLI DNA sequence.

Gene name	DNA Sequence
R.CcoLI	5’-<br>ATGAAATTCAAAATCGATTACGAACTGCCGCTGACCTCAGTGGCAGGCAAA<br>ATCCGTATTAAACAGCGCTCGACCTTCAACGACTACGGTCTGCCGGTTGCT<br>CCGACGAAAATCAACATCAACGTGAAACATTACGTTGAATGGCAAATCGGCT<br>ACGATATGGTCGCGGGCAAAAATGACGGTAACTTTATTGGCGCCAACGGTA<br>AAGATAAAAAACTGTACGAACTGTCCGACATCATCTTCCAGTTTTTCAAACA<br>CAACATCATCCTGAAAGAAAACCTGTTTGGCATCAAAAATTTCCTGGAAAAC<br>AACGAAGAACTGATCGAAGATAAAATGAAAATCAACCGTACCAACTTCACGC<br>AGAAACAAGTTGCGGGTATTAACTTCCTGGAAAGCTATGTCTCTTACCCGCT<br>GCTGGTGTACCAATTCAACAACAACGAATTCCTGAGCGAAATCATCATCAAA<br>GAAAAACAGCGCGCAATCGGCGTTCAGGGTATGCTGTACTTTTGCTTCCCG<br>GTCCATCTGCTGAAAAATATTAACGGCGAACGTAATTTTCTGAACCGCTGTA<br>TCGAAAGTAAAGAAAAAGGTTACCTGGAAATCTCCCGTAACAACATCAACAT<br>TTTTCTGGAAATGCTGAAAATCTTCGGTATTCTGAGCAATAACCACCGCTAC<br>GATGTGCTGCAGATCATTGAATTTATTCTGAATTCTAAATGA-3’

Supplementary Table S2. Primer sequences.

Primer name	Sequence
Cloning-F	5'-TCCCCCGGGATGAAATTCAAAATCGATTACGAACTGC-3'
Cloning-R	5'-CCCAAGCTTTCATTTAGAATTCAGAATAAATTCAATGATCTGC-3'
D53A-F	5’-GGCTACGCTATGGTCGCGGGCAAAAATGAC-3’
D53A-R	5’-GACCATAGCGTAGCCGATTTGCCATTCAAC-3’
N67A-F	5’-GGCGCCGCCGGTAAAGATAAAAAACTGTACGAAC-3’
N67A-R	5’-TTTACCGGCGGCGCCAATAAAGTTACCGTC-3’
K71A-F	5’-CGAACTGTCCGACATCATCTTCCAGTTTTTCAAAC-3’
K71A-R	5’-ATGTCGGACAGTTCGTACAGTTTTGCATCTTTAC-3’
C189S-F	5’-GAACCGCAGTATCGAAAGTAAAGAAAAAGGTTACC-3’
C189S-R	5’-TCGATACTGCGGTTCAGAAAATTACGTTCGC-3’
D225N-F	5'-CCGCTACAATGTGCTGCAGATCATTGAATTTATTC-3'
D225N-R	5'-AGCACATTGTAGCGGTGGTTATTGCTCAG-3'

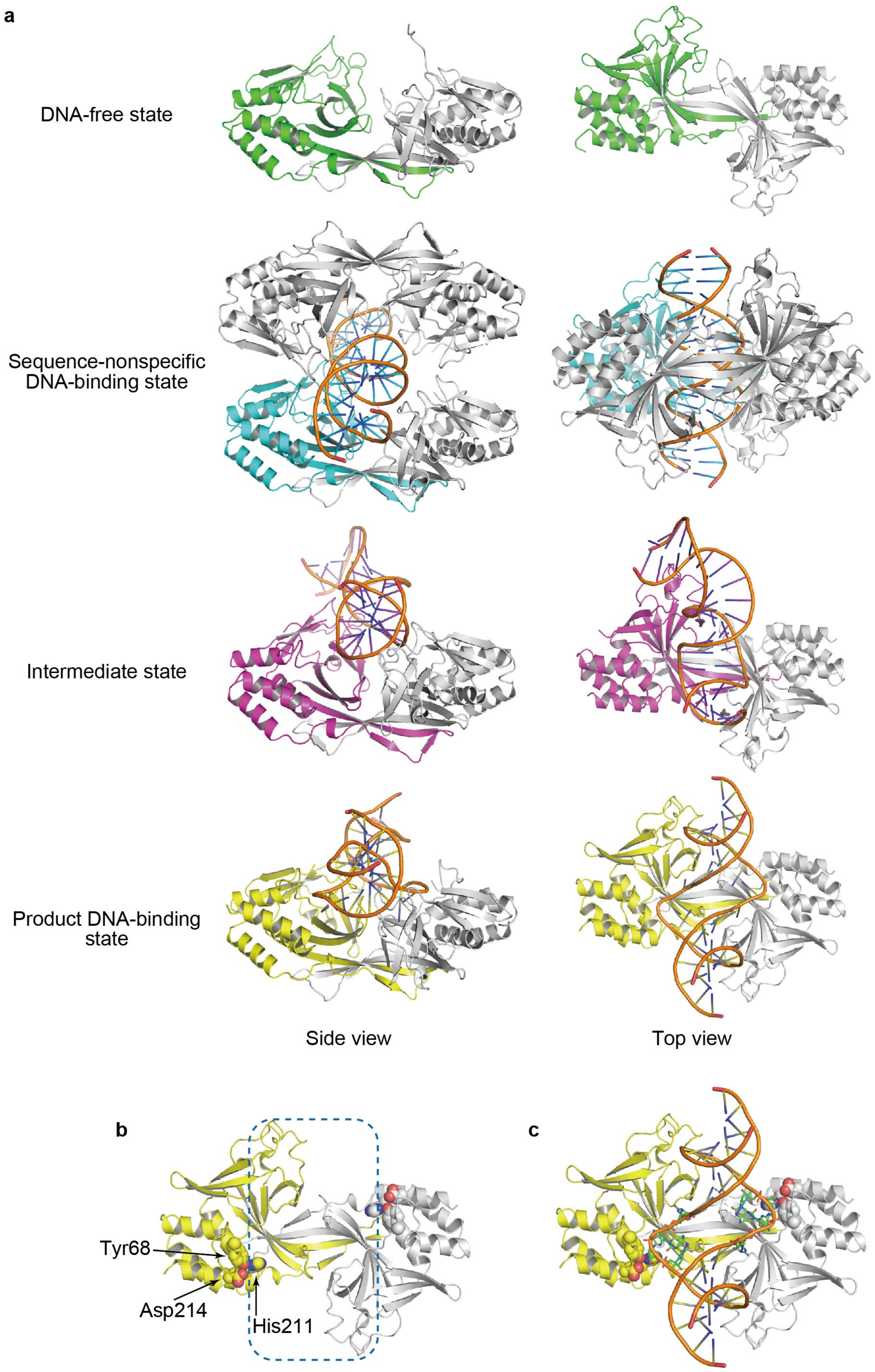
a
DNA-free state
Sequence-nonspecific DNA-binding state
Intermediate state
Product DNA-binding state
Side view
Top view
b
Tyr68
Asp214
His211
c

Supplementary Figure S1. Structures of R.PabI. (**a**) Side and top views of the R.PabI structures. Protomers in the DNA-free state, the sequence-nonspecific DNA-binding state, the intermediate state and the product DNA-binding state are colored green, cyan, magenta, and yellow, respectively. (**b**) The catalytic residues in the product DNA-binding state (Tyr68, His211 and Asp214) are shown by sphere models. The HALFPIPE region of R.PabI dimer is indicated by a blue dotted square. (**c**) The structure of the sequence 5'-GTAC-3' in the product DNA-binding state is shown by green stick models.

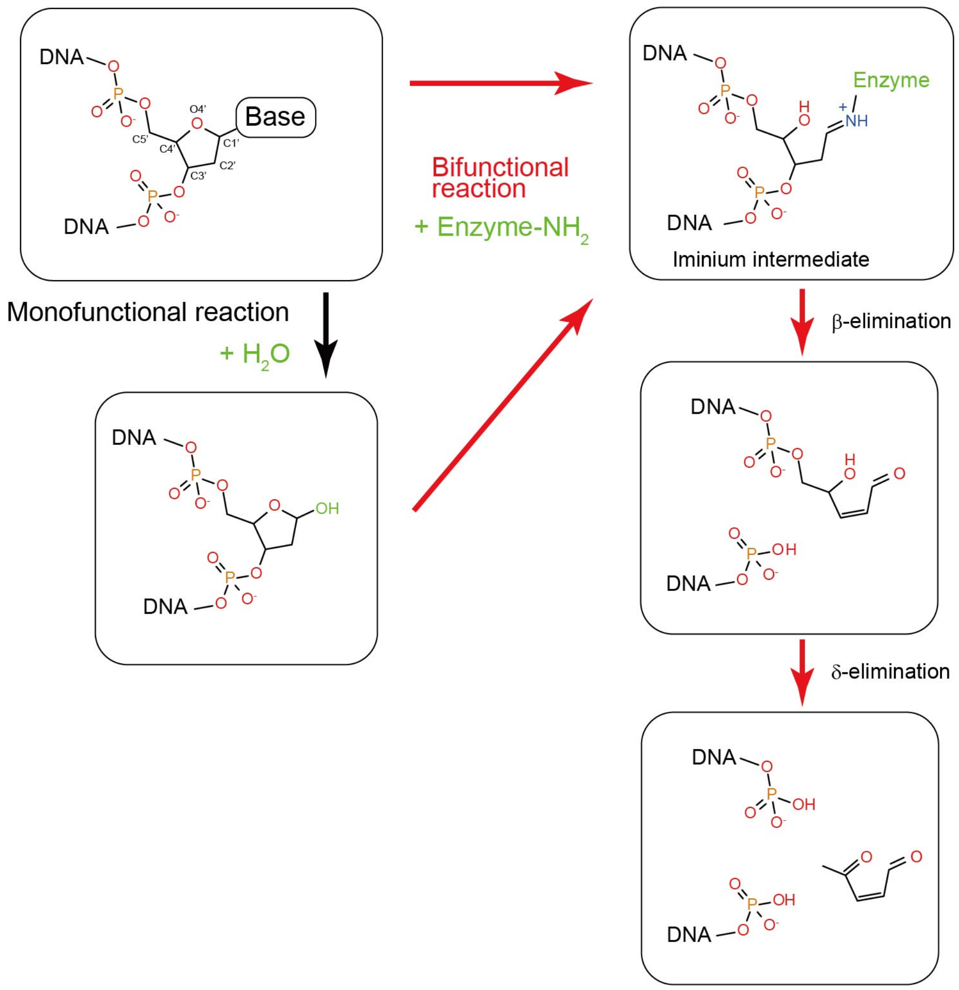

Supplementary Figure S2. DNA cleavage mechanisms of monofunctional and bifunctional DNA glycosylases. A monofunctional DNA glycosylase cleaves an *N*-glycosidic bond by hydrolysis (a black arrow). A bifunctional DNA glycosylase utilizes an amine group to form an iminium intermediate and cleave the DNA backbone by β-elimination (red arrows). Some bifunctional DNA glycosylases also clave DNA by δ-elimination.

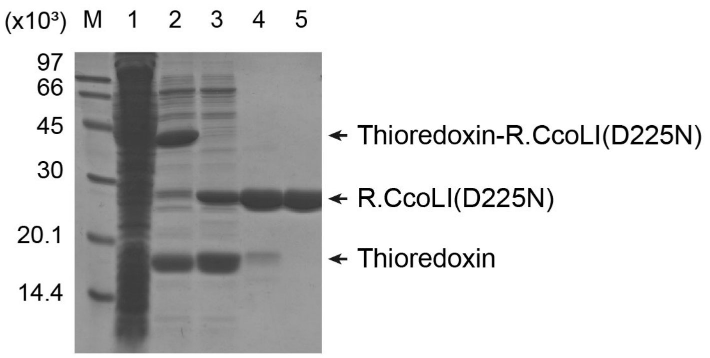

Supplementary Figure S3. Purification of R.CcoLI(D2254N). Lane 1, cell extract of *E. coli* harboring pET48b-R.CcoLI(D225N) plasmid; lane 2, after Ni-NTA column purification; lane 3, after HRV3C treatment; lane 4, after Mono S column purification; lane 5, after gel filtration. The image is a cropped gel image. The full image is shown in Supplementary Figure S7.

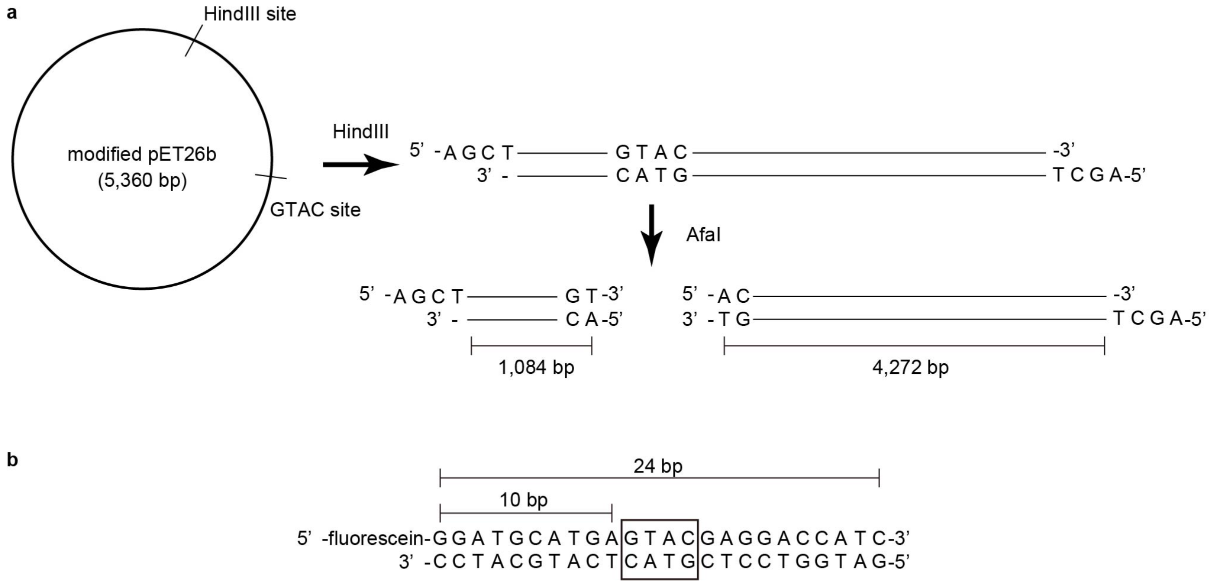

Supplementary Figure S4. DNA substrates. (**a**) A DNA substrate used for the DNA cleavage assay. The linear dsDNA after HindIII treatment contains one 5'-GTAC-3' site and is separated by AfaI and R.CcoLI into two DNA fragments. (**b**) A dsDNA sequence (24 bp) used for the DNA glycosylase assay. The 5'-GTAC-3' site is indicated by a black box.

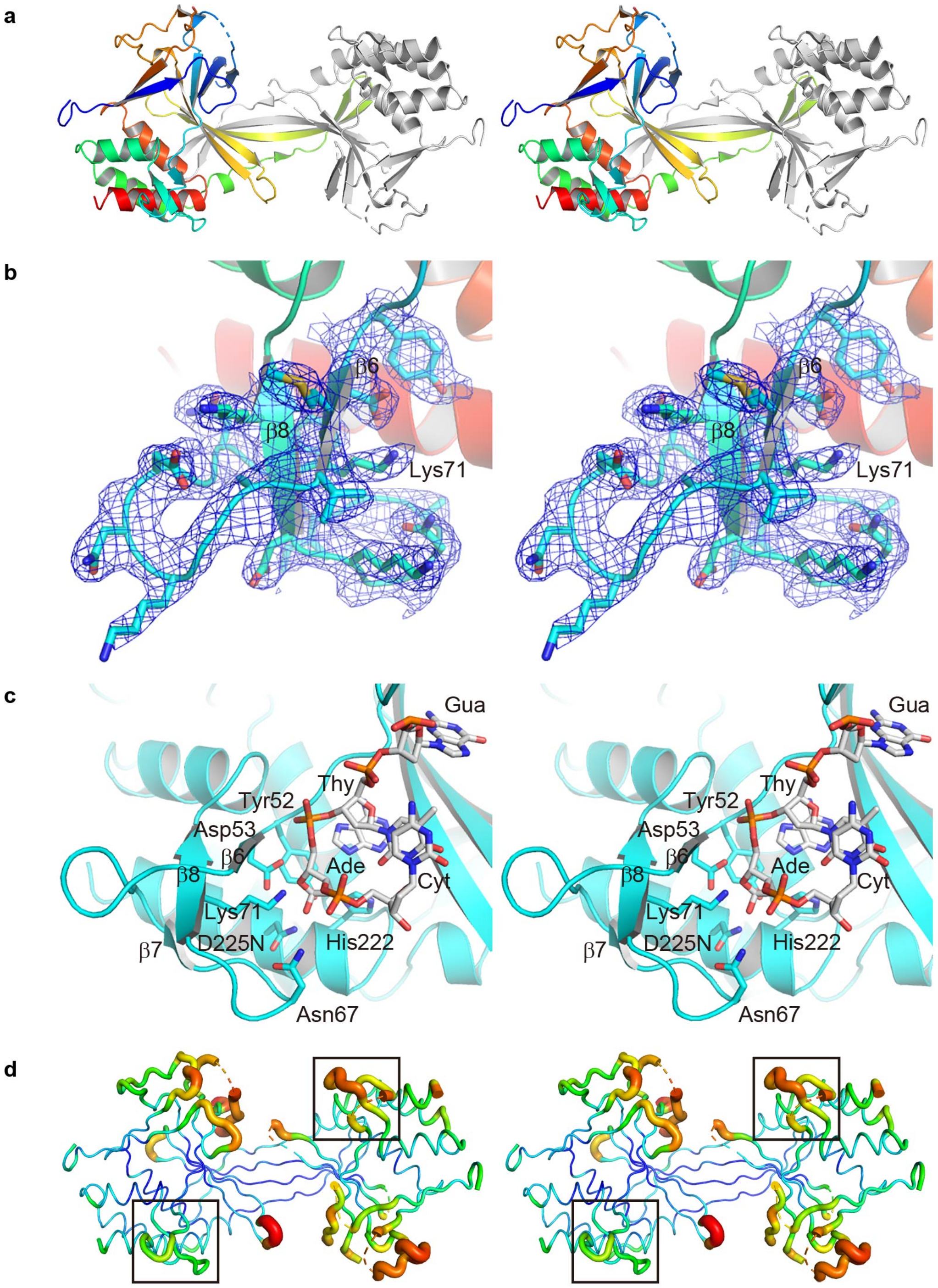

Supplementary Figure S5. Stereo diagrams. (**a**) Stereo diagram of Figure 2a. (**b**) Stereo diagram of the composite omit map (sigma-A weighted $2F_{O}$-F_{C}, 1 σ) around R.CcoLI specific antiparallel β-sheet. (blue mesh) (**c**) Stereo diagram of Figure 4b. (**d**) Stereo diagram of Figure 6b.

a

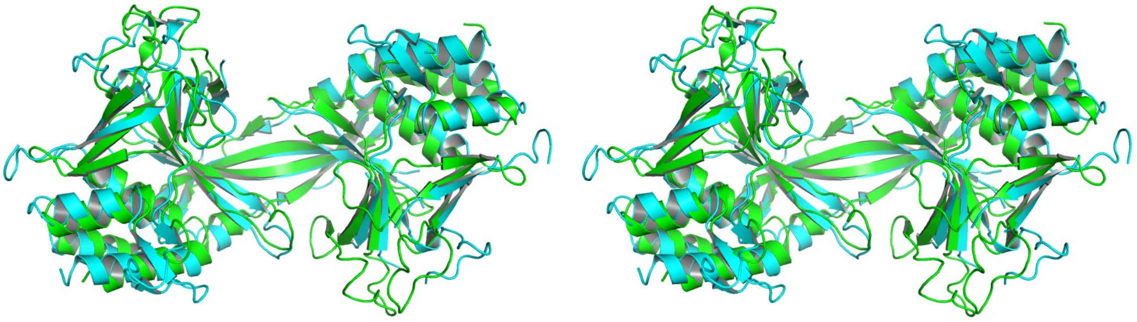

R.CcoLI - DNA free state of R.PabI

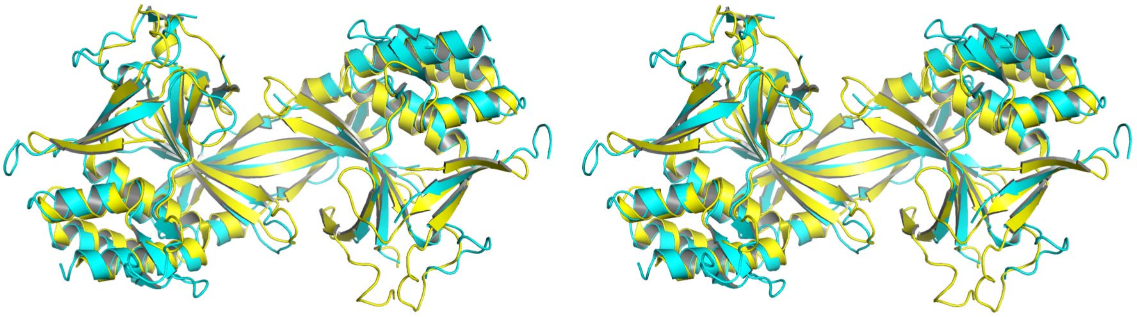

R.CcoLI - sequence-nonspecific DNA-binding state of R.PabI

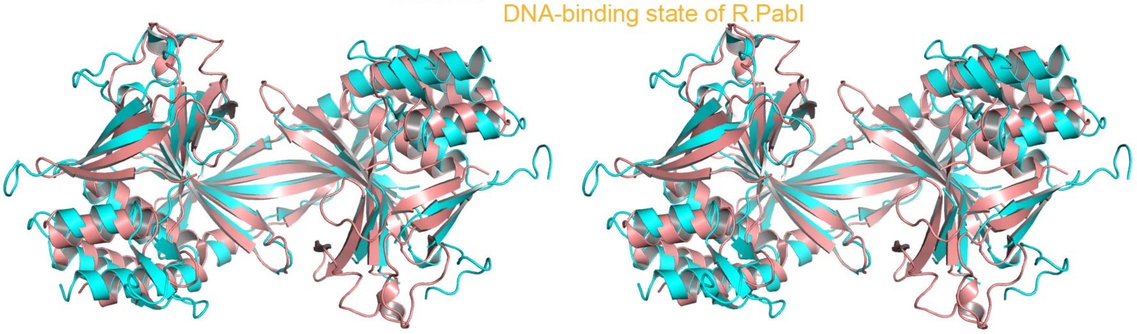

R.CcoLI - intermediate state of R.PabI

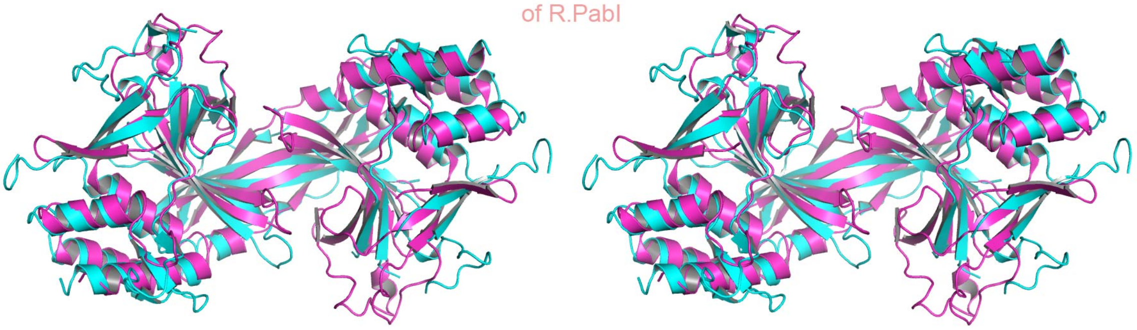

R.CcoLI - product DNA-binding state of R.PabI

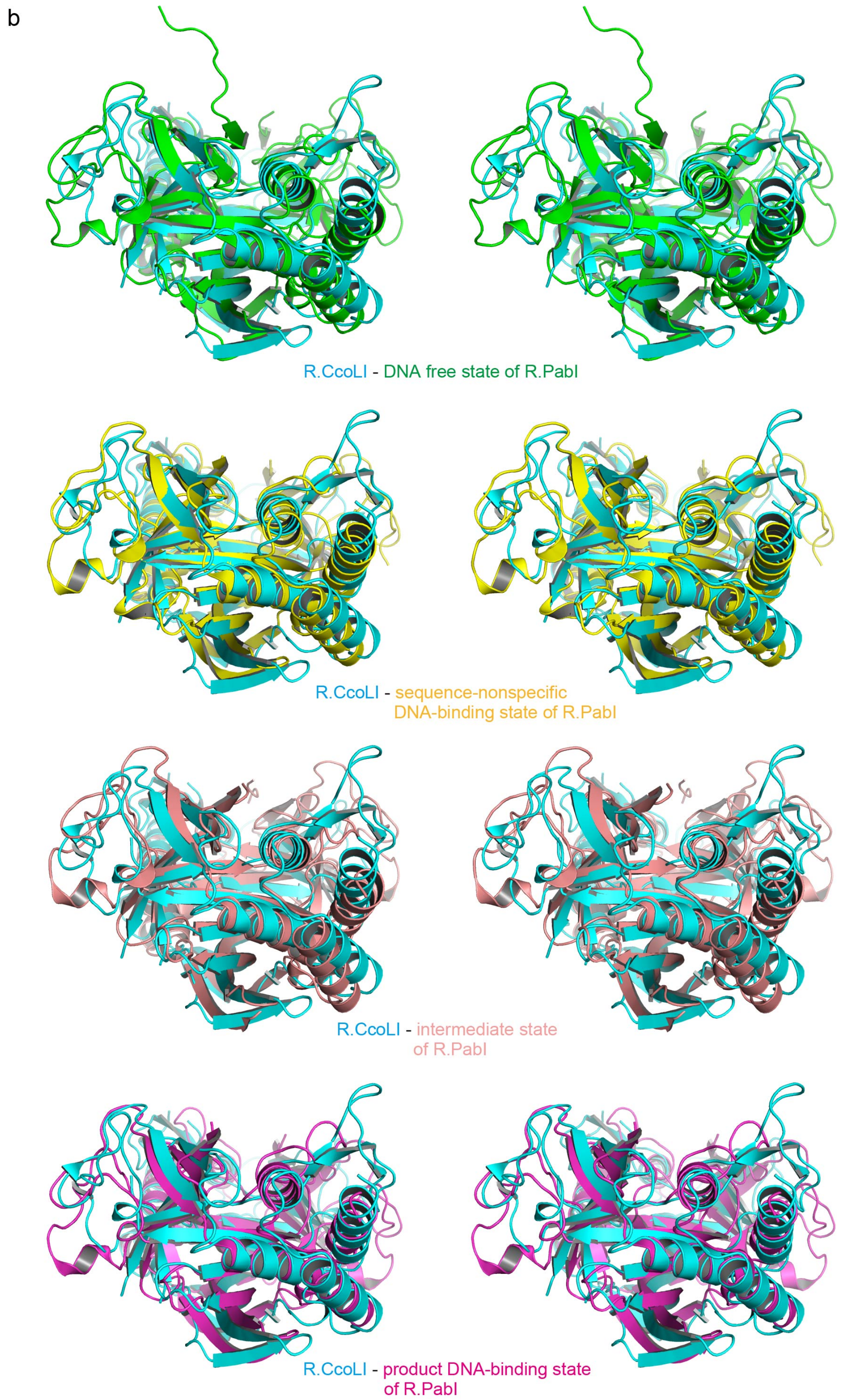

Supplementary Figure S6. Superposition of the dimeric structures of R.CcoLI and R.PabI. (**a**) Stereo diagrams of Figure 3d. (**b**) Stereo diagrams of the side views of Figure 3d.

a

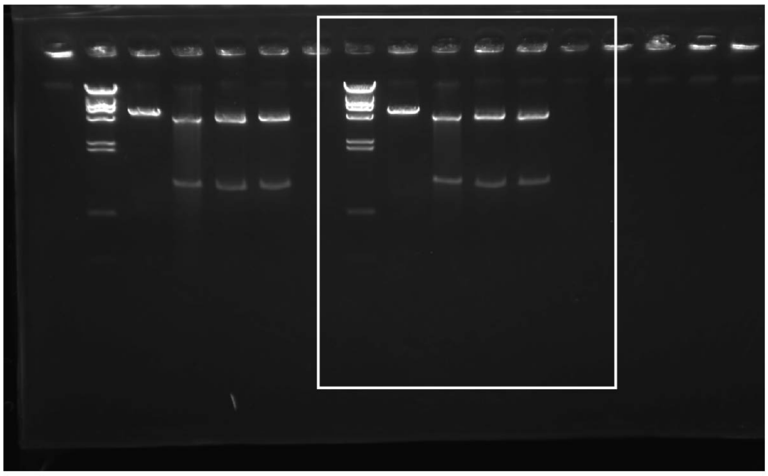

b

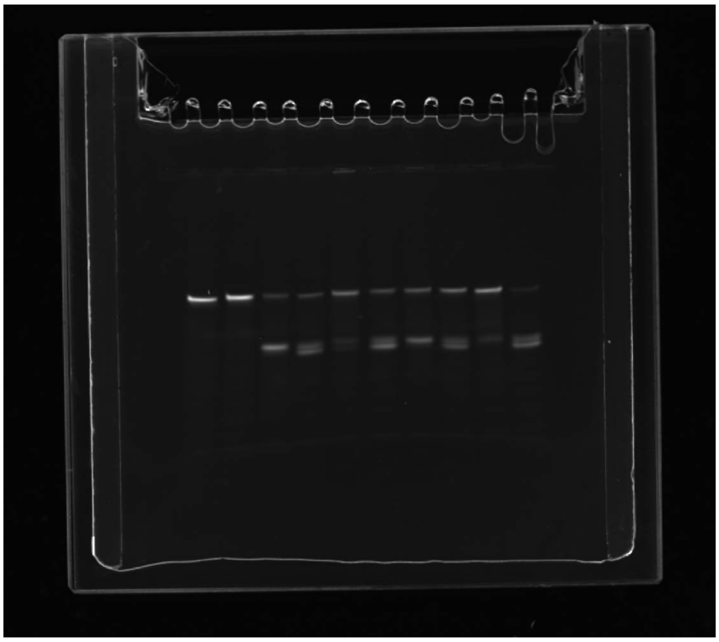

c

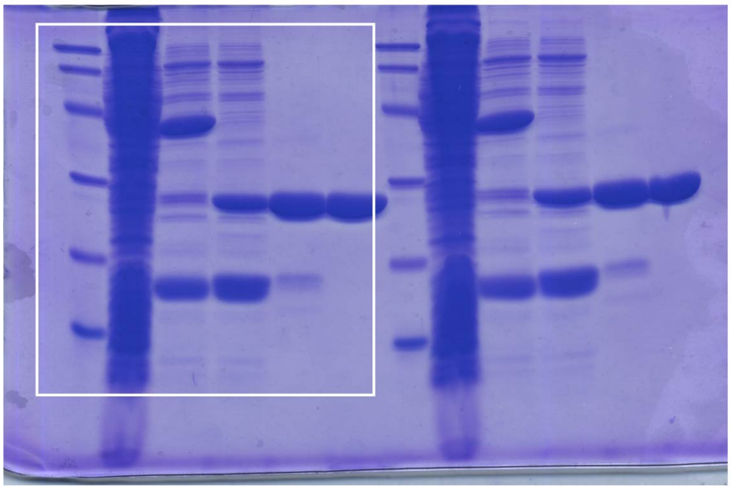

Supplementary Figure S7. Gel images. (**a**) The full gel image of Figure 1d. (**b**) The full gel image of Figure 5a. (**c**) The full gel image of Supplementary Figure S3.
